# Caregiving to Older Adults With a Physical Limitation: Evidence From the Mexican Health and Aging Study

**DOI:** 10.1093/geroni/igac081

**Published:** 2023-01-09

**Authors:** Carlos Díaz-Venegas, Rafael Samper-Ternent, Rebeca Wong

**Affiliations:** Monterrey, Nuevo Leon, Mexico; Department of Management, Policy and Community Health, School of Public Health, the University of Texas Health Science Center in Houston, Houston, Texas, USA; Department of Population Health and Health Disparities and WHO/PAHO Collaborating Center on Aging and Health, the University of Texas Medical Branch, Galveston, Texas, USA

**Keywords:** ADL, Disability, Help, IADL, Mexico

## Abstract

**Background and Objectives:**

Many older adults face physical limitations to performing activities of daily life (ADLs) and instrumental activities of daily life (IADLs) and seek help performing them. In Mexico, family caregivers, especially spouses and adult children, traditionally take care of older adults. However, a detailed characterization of the care received has not been thoroughly provided. We sought to identify socioeconomic, demographic, and health-related differences in receiving help among older adults reporting physical limitations.

**Research Design and Methods:**

Using the 2012 wave of the Mexican Health and Aging Study, we provided information on adults aged 60 and older who reported one or more physical limitations and whether they received help or not. We estimated 2 logistic regression models to obtain the odds ratios (ORs) of receiving help among individuals with an ADL limitation and those with an IADL limitation.

**Results:**

Adults with ADL limitations received, on average, approximately 10.7 hr of assistance per day, whereas those with at least 1 IADL limitation received around 7.7 hr of help per day. Women were more likely to receive help with ADLs than men (OR = 2.35). Individuals with chronic conditions such as hypertension, diabetes, and arthritis also received more help with both ADLs and IADLs.

**Discussion and Implications:**

Our work suggests that help received does respond to the care needs of older adults, but future research should focus on the burden of care for caregivers and expand this analysis using a longitudinal data approach.

**Translational Significance:** Functionally limited older adults in low-middle income countries often receive informal care provided by families, frequently relying on supplementary income programs. Evidence on the socioeconomic and health conditions of those who receive care is a critical stage in developing effective interventions to address the needs of old adults with impairments and thus, to better support aging societies. The study presents evidence of care received by Mexican adults aged 60 or older who report having a limitation with at least one basic activity of daily life or instrumental activity of daily life.

## Background and Objectives

Population aging has taken center stage in the fields of health and sociology, particularly in low and middle-income (LMI) countries, as rapid declines in fertility and mortality rates and an increase in life expectancy have resulted in a larger percentage of the population aged 60 years and older. In Mexico, between 1950 and 2020, life expectancy increased from 48 to 75 years, the fertility rate dropped from 6.8 to 2.1 children, and the mortality rate fell from 18.5 to 6.1 deaths per 1,000 inhabitants (United Nations, 2020). As a result, the proportion of individuals aged 65 or older has dramatically increased from 7.7% in 1950 to 18.0% in 2020 (Gerst-Emerson et al., 2015; United Nations, 2020). However, Mexico still faces challenges, such as a low level of economic development and a lack of proper access to health care and other social services for all. The entire population also faces the burden of noncommunicable chronic conditions in combination with infectious diseases (Samper-Ternent et al., 2012).

Population aging has presented issues for older adults dealing with limitations to their activities of daily living (ADLs), or basic tasks of everyday life (Katz et al., 1963), and instrumental activities of daily living (IADLs), which focus on being independent (Lawton & Brody, 1969). Disability that causes physical limitation is seen as a reversible process that older adults can recover from, either partially or completely (Díaz-Venegas & Wong, 2020), but the topic of an individual receiving help when they have one or more of these limitations has not been fully studied in the context of a LMI country. At age 50, men are expected to spend around 10 years and women around 6 years of their remaining lives with severe disability (Chirinda & Chen, 2017), thus requiring caregiving, most likely from their family members.

Physical limitations and cognitive/mental limitations are the two main reasons for an older adult to need and seek help (National Academies of Sciences: Engineering and Medicine, 2016). In Latin America, more than 8 million older adults need help with their basic care. Long-term care services are scarce, and families provide most of the care with limited support and resources (Cafagna et al., 2019). Mexico is no exception to this reality and economic factors influence the bulk of familial decisions (Orozco-Rocha et al., 2021) and dependent older adults face high overall costs of caregiving (Gutiérrez-Robledo et al., 2022). Even though the entire population has access to health care in theory, long-term care programs that target older adults are not integrated in the health care system, and there is limited availability (Giraldo-Rodríguez et al., 2019). Hence, families rely on federal or state supplemental income programs that give extra money to adults aged 65 and older to compensate for the income lost by the caregivers inside the family.

These benefits began in Mexico City in 2001 and then expanded to the federal level, reaching rural areas in 2007 and countrywide in 2013 (Aguila et al., 2016). This program has led to some poverty reduction, better mental health and fewer depressive symptoms among older adults, and more integration inside the household by older adults participating in familial activities and sometimes even helping in taking care of their grandchildren (Galiani et al., 2016). With the lack of long-term care programs, however, this is the only available way for families to relieve some of the burdens of caregiving (Aguila et al., 2019; Flores-Martínez & Garay-Villegas, 2019).

Most of the help given to older adults is by family caregivers, with spouses and adult children being at the top of the list. Cultural norms have placed the women as the preferred caregiver, and in most cases, they also care for their own children and do other household-related activities (Comisión Económica para América Latina y el Caribe, 2021; Garay-Villegas et al., 2020; López-Ortega & Aranco, 2019; ONU Mujeres, 2018) and caregiving is seen as an unproductive activity where specific skills are not usually needed (OXFAM, 2020). In fact, women tend to do around 76% of the unpaid care work, spend 3.2 times more time providing care than men and, as a result, tend to have fewer years of schooling and are more likely to be unemployed (Aguila et al., 2019; Angst et al., 2019; International Labor Organization, 2018). These economic and social inequalities surrounding caregiving tend to put women at a disadvantage with respect to their physical and emotional health (Arroyo et al., 2021; Bhan et al., 2020). In addition, sociodemographic characteristics of caregivers and the associated burden of caregiving tend to differ based on the type of disability suffered by the older adult and the economic environment, as evidenced in examples of older adults with dementia in Australia (Connors et al., 2020), older adults with a stroke in São Paulo, Brazil (Caro et al., 2018) and older adults with severe mental illness in several sub-Saharan countries (Addo et al., 2018).

Informal care is known to bring both positive (Boerner et al., 2004; Evercare and National Alliance of Caregiving, 2008) and negative consequences to caregivers and older adults (Beach & Schulz, 2017; De Valle-Alonso et al., 2015; Swartz & Collins, 2019). Previous literature has focused on a conceptual framework of caregivers’ experiences, including the stresses and burdens of caregiving (Lawton et al., 1991; Pearlin et al., 1990; Sörensen et al., 2006). Work in Mexico and Latin America has focused on expanding the scope on caregiving studies and policies to include the cost and implication of providing care for older adults for both the person providing the care and the adult receiving the care. Many argue that the lack of focus on a life course and gender perspective has led to a narrow focus on caregiving with important social and health implications (Garay-Villegas et al., 2021; Rea-Ángeles et al., 2021).

More recent research has explored a proposed framework that integrates concepts from previous models, such as stressors (for both the caregiver and the elder) and mediators, such as the quality of the relationship between the caregiver and the recipient to measure caregiver burnout and general outcomes for both (Gérain & Zech, 2019). Similarly, another proposed framework aims to focus on the older adults and how they are affected by informal caregiving, considering the type of caregiver, the nature of caregiving relationships, and the activities performed during caregiving, in order to measure health-related outcomes for the older adult (Cho, 2007).

One common factor of these two proposed frameworks is their inclusion of background information, socioeconomic and demographic characteristics, of both the caregiver and the recipient. Therefore, we focus on this aspect by providing evidence of care received by adults aged 60 or older who report having a limitation to at least one ADL or IADL and examining the socioeconomic and health-related covariates that influence whether the older adult receives help. This work fills a gap in the literature regarding the socioeconomic and demographic characteristics of older adults (e.g., age, gender, and place of residence) who receive help for a physical limitation in the context of a national sample of adults in a LMI country. Similarly, the impact of specific comorbidities on older adults’ need for help has been researched in developed nations (Covinsky et al., 2003; Han & Haley, 1999), but such research is lacking in LMI countries. Thus, the objective of this study was to determine whether there are socioeconomic, demographic, or health differences in patterns of receiving help among older adults with difficulty performing daily activities.

## Research Design and Methods

The data were gathered from the Mexican Health and Aging Study (MHAS), a nationally representative panel investigation of health and aging in Mexicans born in 1951 or earlier. The baseline data, consisting of 15,186 in-person interviews, were collected in 2001 with follow-ups in 2003, 2012, 2015, and 2018. We focused on the 2012 wave only as a new sample was added for this wave so that the survey remained representative of community-dwelling adults aged 50 or older. Further information about the MHAS is available elsewhere (Wong et al., 2015). This study was approved by the Institutional Review Board or Ethics Committee of the University of Texas Medical Branch in the United States as well as the Instituto Nacional de Estadística y Geografía (INEGI) and the Instituto Nacional de Salud Pública (INSP) in Mexico.

### Outcome Variable

The MHAS questionnaire asked the respondents if, because of a health issue, they have difficulty performing several activities, excluding those difficulties believed to last less than 3 months. We combined this question with one asking whether the respondent needed help (from a person or special equipment) to perform this activity. The result was a dichotomous variable for each of the five ADLs (eating, bathing, getting dressed, transfer in/out of bed, and using the toilet) and four IADLs (shopping, managing money, taking medications, and preparing meals) that combines the inability to perform and the need for help. For the 2012 cross-section, we have an eligible sample of 8,835 older adults aged 60 or older.

### Covariates

We extracted or constructed the following variables using data from 2012. Age was measured as a continuous variable. Rural or urban residence was measured with a dichotomous variable as a respondent living in a community with fewer than 100,000 inhabitants (= 1) or not (= 0), respectively. Marital status was measured as a dichotomous variable indicating whether the respondent was married (= 1) or single, divorced, widowed, or separated (= 0). The total cognition score (0–88 points) was the sum of the scores of different tasks measuring visuospatial ability (0–6 points), visuospatial memory (0–6 points), verbal learning (0–8 points), verbal recall (0–8 points), and visual scanning (0–60). For detailed information on each of the tasks, please refer to Michaels-Obregón et al. (2014). In the logistic regression, these scores were presented in quartiles. This is a common statistical method of summarizing the data and splitting it into four equal-sized groups when data is not symmetrically distributed or has some outliers (Dekking et al., 2005), and this method is commonly used for socioeconomic and health-related variables such as cognition scores. See, for example, Fluharty et al. (2021).

The number of depressive symptoms was expressed as a dichotomous variable to capture whether the respondent reported an elevated number of symptoms (CES-D score ≥ 5); the threshold was based on previous literature and validated for the MHAS in a study of 199 older adults at the Salvador Zubirán National Institute of Medical Sciences and Nutrition (Aguilar-Navarro et al., 2007). Self-rated health (SRH) was scored to indicate poor SRH (= 1) and all other categories (excellent, very good, good, fair = 0). Self-reported diagnoses of hypertension, diabetes, cancer, respiratory problems, heart conditions, or stroke were captured by a dichotomous variable for each to observe differences in caregiving for each chronic condition. Average individual monthly income was expressed as three separate dichotomous variables: indebted or no income (reference), less than 5,000 Mexican Pesos (MXP), and 5,000 MXP or more (1 USD is equivalent to approximately 20.00 MXP at current exchange rates). We used the income values imputed by the MHAS following a similar method as that of the U.S. Health and Retirement Study based on a bracketed unfolding technique to reduce nonresponse and a method of regression sequencing with a SAS-based software (IVEware). More details on the full process are available in the Mexican Health and Aging Study (2016). Health insurance coverage was measured as any type (= 1) or not covered (= 0). Finally, we included gender, measured by male (= 0) or female (= 1), and years of education, indicated by three dichotomous variables: 0 years of formal education (reference), 1–6 years of education, and 7 or more years of education.

We presented descriptive results for those who reported an ADL or IADL limitation stratified by whether they received help or not. Then, we used two logistic regressions to determine the odds ratios of receiving help due to an ADL or IADL limitation after controlling for the covariates listed earlier. We used Stata 15.1 for the analysis (StataCorp, 2015).

## Results

**Figure 1.**
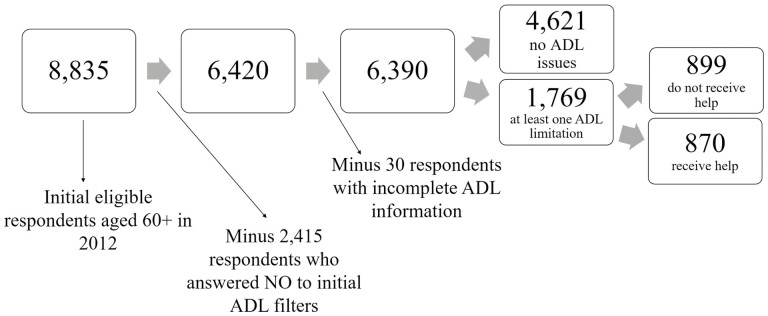
Data flow chart for activities of daily living (ADLs). Source: Author’s own elaboration of data from the Mexican Health and Aging Study (2012).

**Figure 2.**
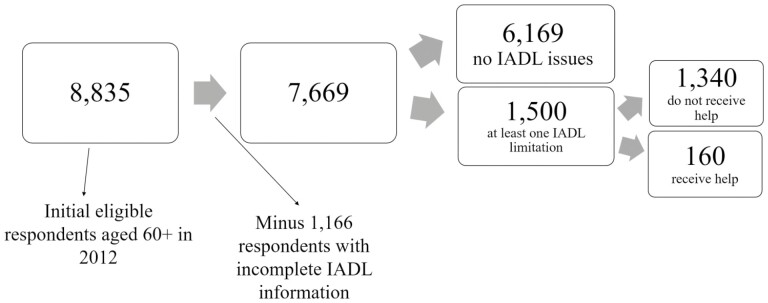
Data flow chart for instrumental activities of daily living (IADLs). Source: Author’s own elaboration of data from the Mexican Health and Aging Study (2012).

To understand where our sample comes from, we included data flow charts in Figure 1 (for ADLs) and Figure 2 (for IADLs). In Figure 1, respondents went through a preliminary series of 14 filter questions to determine if they had limitations in performing some basic activities. If they answered “no” to all of them, then they were not asked the questions about limitations to the 5 ADLs. We ended up with 6,390 respondents, of which 1,769 (27.7%), aged 60 or older, had difficulty performing at least one ADL. Of these 1,769 respondents, 899 did not receive any help, and 870 did receive help. Physical difficulties with walking (53%), transferring into/out of bed (52%), and going to the toilet (40%) were the most prevalent. For respondents who received help, the MHAS included additional questions about the caregiver’s identity. The respondents were asked about the person who most often helps them with ADLs and/or IADLs, and they could list as many caregivers as they wanted with their names, relationship to them, and how many hours per day and days per month they were receiving help. For ADLs, on average, older adults received care for approximately 10.7 hr per day and 23.3 days per month. Stratified by gender, men received approximately 11.0 hr per day and 23.3 days per month of care, and women received an average of 10.4 hr per day and 23.2 days per month of care (results not shown).

The respondents included in Figure 2 did not have any preliminary filter; thus, we ended up with 7,669 respondents, of which 1,500 (19.6%), aged 60 or older, reported difficulties with performing at least one IADL. Of these 1,500 respondents, 1,340 (89%) did not receive any help, and only 160 (11%) did receive help. Shopping (69%) and preparing meals (35%) were the IADLs with the highest percentages of individuals reporting difficulties. For the small share who received IADL help, this help was, on average, approximately 7.7 hr per day and 23.3 days per month. Separating these numbers by gender, men received help for approximately 8.2 hr per day and 23.2 days per month, whereas women received care for approximately 7.3 hr per day and 20.6 days per month (results not shown).

Table 1 presents descriptive information about the 1,769 respondents who had problems with at least one ADL and the 1,500 respondents who had problems with at least one IADL, stratified by whether they received help. Regarding ADLs, respondents who did not receive help were, on average, 5 years younger (73.8 vs 78.9 years) and more likely to be married (55.9% vs 37.6%) and presented some contrasts in terms of chronic conditions compared with those who received help. Those who did not receive help were more likely to report hypertension (63.5% vs 55.1%) and cancer (5.2% vs 3.5%) but less likely to report respiratory diseases (7.4% vs 10.3%) and stroke (5.0% vs 12.3%). Those who did not receive help also had higher total cognition scores compared with those who received help (30.6 vs 28.7 points). In terms of specific ADL limitations, respondents who did not receive help reported having difficulty transferring into/out of bed (50.2%). In contrast, almost two thirds of the respondents who did receive help reported having difficulties bathing (64.1%), transferring into/out of bed (62.4%), or dressing (58.6%; results not shown).

**Table 1.** Characteristics of Mexican Adults Aged 60 or Older by Type of Limitation and Help Received

Variables	ADLs				IADLs			
	No help (*n* = 899)		Help (*n* = 870)		No help (*n* = 1,340)		Help (*n* = 160)	
	*M*	%	*M*	%	*M*	%	*M*	%
Age in years***	73.8		78.9		70.6		75.5	
Female		61.7		69.7		61.9		52.4
Years of education	2.8		3.0		2.3		3.2	
Married***		55.9		37.6		64.9		49.4
Poor self-rated health**		37.2		48.0		32.2		33.6
Hypertension		63.5		55.1		52.5		56.4
Diabetes**		28.9		33.6		26.4		29.4
Cancer		5.2		3.5		1.4		3.3
Respiratory diseases		7.4		10.3		11.6		7.9
Stroke*		5.0		12.3		5.7		5.7
Heart conditions		7.2		4.9		6.5		5.8
CES-D score	5.1		5.6		4.9		4.6	
Total cognition score	30.6		28.7		29.6		28.9	

*Notes*: CES-D = Center for Epidemiologic Studies Depression Scale. Authors’ own elaboration of data from the Mexican Health and Aging Study (2012). Values shown are unweighted. Chi-square tests were calculated to establish whether the differences between those who received help versus those who did not receive help were statistically significant for each covariate in activities of daily living (ADLs) and, separately, in instrumental activities of daily living (IADLs). The asterisks next to each covariate indicate that there were statistically significant differences between those who received help and those who did not in both ADLs and in IADLs.

*p ≤ .05; **p ≤ .01; ***p ≤ .001.

Regarding IADLs, compared with respondents who received help, those who did not receive help were, on average, 5 years younger (70.6 vs 75.5 years) and more likely to be married (64.9% vs 49.4%) and had a similar prevalence of chronic conditions except for respiratory diseases (11.6% vs 7.9%) and cancer (1.4% vs 3.3%). In terms of specific IADL limitations, respondents who did not receive help reported having difficulty shopping (40.7%). Respondents who did receive help reported difficulties with shopping (71.0%) and preparing meals (58.2%; results not shown).

Next, we estimated two logistic regressions to obtain the odds ratios of receiving help among (a) those with an ADL limitation and (b) those with an IADL limitation. In each regression, we controlled for the independent variables described in the previous section introduced sequentially in three models. Model 1 controlled for age, gender, and educational attainment. Model 2 introduced average monthly income, insurance coverage, and health and chronic conditions. Model 3 introduced the total cognition score in four quartiles and the dummy variable for excessive depressive symptoms.

**Figure 3.**
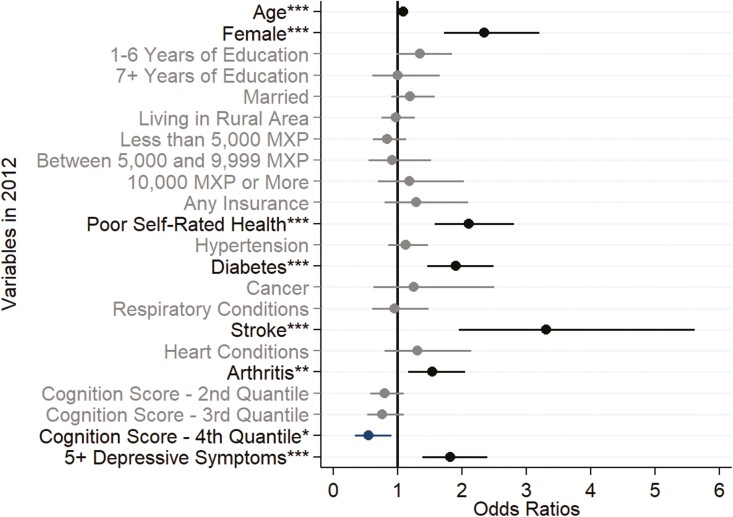
Odds ratios of receiving help due to a limitation in at least one activity of daily living (ADL) among Mexican adults aged 60 or older in 2012. Source: Author’s own elaboration of data from the Mexican Health and Aging Study (2012). Note: Variables in bold are statistically significant. Confidence intervals are shown for each variable. *p ≤ .05; **p ≤ .01; ***p ≤ .001.

To summarize the regression results, Figure 3 shows the odds ratios for receiving help for an ADL limitation according to Model 3. Older Mexican women were over two times more likely to receive help than older men when facing an ADL limitation (OR = 2.35). Socioeconomic conditions such as educational attainment and average monthly income were not statistically significant predictors for receiving help. In contrast, respondents who self-rated their health as “poor” were more likely to receive help than those who did not (OR = 2.10). Some chronic conditions also drastically increased the odds of receiving help for those with an ADL limitation, such as diabetes (OR = 1.91), stroke (OR = 3.31), and arthritis (OR = 1.54). Furthermore, the better an individual’s cognitive function (higher quantile), the lower their odds ratio of receiving help (OR fourth quantile = 0.55).

**Figure 4.**
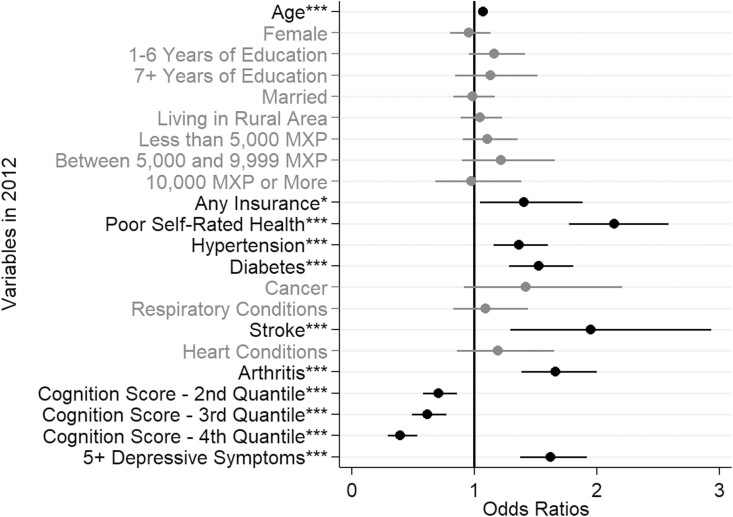
Odds ratios of receiving help due to a limitation in at least one instrumental activity of daily living (IADL) in Mexican adults aged 60 or older in 2012. Source: Author’s own elaboration of data from the Mexican Health and Aging Study (2012). Note: Variables in bold are statistically significant. Confidence intervals are shown for each variable. *p ≤ .05; **p ≤ .01; ***p ≤ .001.

Figure 4 shows the odds ratios for receiving help for an IADL limitation using Model 3. In contrast to the results for ADLs, gender does not seem to have played a significant role in determining the odds of the respondent receiving help due to an IADL limitation. The health and chronic condition variables remain at the forefront of our regression results that increased the odds of receiving help. Respondents who self-rated their health as “poor” compared to all other options were more likely to receive help (OR = 2.14), and nearly all chronic conditions, except for the heart and respiratory conditions and cancer, increased the likelihood of the respondent receiving help due to an IADL limitation, with stroke being the highest (OR = 1.93). Additionally, all quartiles of the total cognition score were statistically significant; the higher quartiles, representing higher cognitive function, were associated with lower odds of receiving help due to an IADL limitation (OR fourth quantile = 0.39).

## Discussion and Implications

We used a national sample of Mexican adults aged 60 or older to determine the patterns of ADL and IADL limitations and document key socioeconomic, demographic, and health-related characteristics of those who receive help because of these limitations. We estimated logistic regression models to examine how these covariates are associated with individuals’ odds of receiving help due to a limitation. Our results show that older Mexican women were more likely to receive help than older men, and that the presence of chronic conditions such as hypertension, diabetes, stroke, and arthritis are highly pertinent to receiving care for both ADLs and IADLs. These results highlight the role chronic conditions play as each one brings a different set of issues for the caregiver and the rest of the household to deal with, such as the type of chronic condition, the severity of the condition, the cost of treatment, the socioeconomic resources (education and wealth) available in the household, the time needed to provide care to the older adult, and the potential labor force participation exit or reduction of time at work leading to a decrease in income (Quashie et al., 2021; Schofield et al., 2014).

Our results also align with previous studies that used a U.S. sample in a similar period (National Academies of Sciences: Engineering and Medicine, 2016), and two using Mexican data showing women as the main caregivers (INEGI & INMUJERES, 2019) and women spending around 14 hr per day taking care of a dependent (Salazar-Barajas et al., 2019). However, there are differences in the way caregiving is measured and the reference period for receiving help which tend to make comparisons difficult even among quantitative studies (Schulz et al., 2020).

We further described the intensity of the care provided using a nationally representative sample of noninstitutionalized Mexican adults. Although this was not the main objective of our manuscript, it is important to document this information as it can indicate the burden on caregivers (Bose et al., 2021; Potter, 2018). The intensity of care received seems greater compared with that reported in previous studies of Hispanics in the United States, who received an average of 11 hr of care per week (Mehdipanah et al., 2022; Reuben et al., 2021).

For older adults with functional limitations in Mexico, the network of caregivers provides, on average, almost 8 hr of care per day during 23 days of each month, which is equivalent to a full-time job. It has been established that the burden of caregiving has both economic and health implications as well as positive and negative affects on those who provide care (Moraes-Balbim et al., 2020; Roth et al., 2015). These aspects must be considered in future studies to measure the consequences of caregiving and develop interventions to educate and support caregivers. Our study was an initial step toward a better understanding of critical aspects of caregiving in LMI countries, such as Mexico, with a considerable prevalence of informal arrangements for this care, and future work will explore additional aspects of caregiving that may affect—positively or negatively—both care recipients and their informal caregivers.

Our work has some limitations. First, we only used data for 2012 as the MHAS introduced a new sample of older adults to preserve the nationally representative nature of the study. This meant having a larger pool of adults 60 and older in our analysis, but we did not conduct a longitudinal analysis that would provide long-term information on caregiving and caregivers in Mexico. Second, several of our variables relied on self-reported information, such as self-rated health and chronic conditions, which may potentially affect our results. Third, we have a considerable amount of missing IADL information. This may be due to the gendered nature of activities; for example, older Mexican women tend to handle activities like going shopping, whereas older Mexican men handle money management. Finally, we found differences in the number of older adults who received or did not receive help if they had a chronic condition. Previous literature has shown that older adults with chronic condition are more likely to report a perceived unmet health care need (Jang et al., 2021). However, the MHAS does not include follow-up questions to identify the reasons why these needs go unattended.

Our work examining the help received by older adults seems to indicate that help responds to individuals’ care needs with respect to both physical and cognitive function. However, further research should consider the socioeconomic and demographic characteristics of the individuals in the caregiving network, usually family members, and examine their respective burdens of care. These individuals likely need to balance their family responsibilities to accommodate providing care to the older adult and, in some cases, participate in the labor market to earn income. Moreover, future research should consider the consequences of the decisions made by caregivers regarding the care of their older family members and the nature of the interactions between the caregiver and the older adult receiving care. Finally, research should focus on older adults who do not receive help despite reporting difficulties with performing an ADL or IADL. The consequences of and reasons behind the lack of help should be carefully examined.

As population aging continues to advance in LMI countries, our research attention should focus on building major health programs that target older adults, but informal caregiving must be integrated into this research agenda. Public policy should be able to address different factors, such as the economic burden faced by caregivers and the health outcomes faced by the older recipients of care and recognize the relevance of informal caregiving in the context of a LMI country like Mexico (López-Ortega et al., 2013). Furthermore, public policies should be designed to not only identify and help older adults who receive help but also identify informal caregivers and examine the unmet needs of older adults who do not receive help.

The Mexican government has not integrated informal care into their policies. Specific programs must be implemented to support the informal caregiver, especially those who also work, in order to combine their work life with the caregiving they provide to avoid the potential for being burnt out (Hurtado-Vega, 2021). One example is the series of courses to promote healthy aging developed by the Mexican National Institute of Geriatrics. One course centers on informal caregivers and teaches basic concepts for proper caregiving in four modules lasting 10 hr per week each (Instituto Nacional de Geriatría, 2022). The federal government also needs to provide relief to informal caregivers. Adult daycare centers and respite care programs that provide temporary relief for caregivers while guaranteeing adequate care to older adults in need (Min et al., 2021; Vargese et al., 2019) have been used successfully in many countries and proposed in Mexico. Federal programs like these can be evaluated and tailored for each state to promote healthy aging targeting specific environmental and socioeconomic conditions.
